# Upregulation of Netrin-1 in the hippocampus mediates the formation of visceral hypersensitivity induced by maternal separation

**DOI:** 10.3389/fnmol.2022.908911

**Published:** 2022-07-28

**Authors:** Junwen Wang, Guangbing Duan, Tingting Zhan, Zhiyu Dong, Yan Zhang, Ying Chen, Huihui Sun, Shuchang Xu

**Affiliations:** Department of Gastroenterology, Tongji Institute of Digestive Diseases, Tongji Hospital, School of Medicine, Tongji University, Shanghai, China

**Keywords:** visceral hypersensitivity, maternal separation, Netrin-1, DCC, GluA1, hippocampus

## Abstract

Early adverse life events (EALs), such as maternal separation (MS), can cause visceral hypersensitivity, which is thought to be a key pathophysiological mechanism of irritable bowel syndrome (IBS). Previous studies mainly focused on EALs-induced visceral hypersensitivity in adulthood but did not consider that it may have occurred in the preadult period. We previously found that rats who experienced MS suffered from visceral hypersensitivity starting from the post-weaning period. Moreover, the hippocampus is considered to be critical in regulating the formation of visceral hypersensitivity induced by MS. But the underlying mechanisms throughout different life periods are unclear. In this study, behavioral tests, RNA-seq, lentiviral interference, and molecular biology techniques were applied to investigate the molecular mechanism in the hippocampus underlying MS-induced long-lasting visceral hypersensitivity. It was found that both visceral sensitivity and anxiety-like behaviors were significantly increased in MS rats in post-weaning, prepubertal, and adult periods, especially in the prepubertal period. Subsequently, RNA-seq targeting the hippocampus identified that the expression level of Netrin-1 was significantly increased in all periods, which was further confirmed by quantitative real-time PCR and Western blot. Knocking-down hippocampal Netrin-1 in the post-weaning period by lentivirus interference alleviated visceral hypersensitivity and anxiety-like behaviors of MS rats in the later phase of life. In addition, deleted in colorectal cancer (DCC), instead of neogenin-1(Neo-1) or uncoordinated (UNC5), was proved to be the specific functional receptor of Netrin-1 in regulating visceral hypersensitivity, whose upregulation may result in the most severe symptoms in the prepubertal period. Furthermore, the activation of the Netrin-1/DCC pathway could enhance long-term potentiation (LTP) in the hippocampus, probably *via* recruitment of the AMPA receptor subunit GluA1, which finally resulted in the formation of visceral hypersensitivity. These novel findings suggest that long-lasting over-expression of Netrin-1 can mediate visceral hypersensitivity and anxiety disorder from the post-weaning period to adulthood by activating DCC/GluA1 pathway in the hippocampus. Moreover, early intervention of Netrin-1 in the post-weaning period could lead to significant symptom relief afterward, which provides evidence that the Netrin-1/DCC/GluA1 signaling pathway may be a potential therapeutic target for the treatment of visceral hypersensitivity in clinics.

## Introduction

Irritable bowel syndrome (IBS) is a common disorder of gut–brain interaction, characterized by abdominal pain associated with altered bowel function (Drossman, 2016; Enck et al., 2016). The global prevalence rate of IBS ranges from 3.8 to 4.8%, according to Rome IV criteria (Oka et al., 2020). This causes considerable social–economic issues and affects individual life quality (Black and Ford, 2020; Oka et al., 2020; Palsson et al., 2020). Visceral hypersensitivity is regarded as a principal contributor to the generation of chronic visceral pain in IBS (Simren et al., 2018), but its pathogenesis is still unknown. Early adverse life events (EALs), such as maternal separation (MS), can facilitate pain perception and sensitize pain pathways, leading to a feed-forward cycle promoting chronic visceral sensation disorders (Greenwood-Van Meerveld and Johnson, 2017). However, previous animal studies of EALs-related visceral hypersensitivity mainly focused on adulthood and did not consider that it may have occurred in the preadult period (Park et al., 2016; Liu et al., 2017; Liu et al., 2017; Fuentes and Christianson, 2018). Our previous study has found that rats who experienced MS have suffered from visceral hypersensitivity since the post-weaning period (Yi et al., 2017). It was revealed that EALs-related IBS is hard to treat in adult patients (Barreau et al., 2007; Drossman, 2016). Therefore, to search for a therapeutic target and better strategy for the treatment of EALs-related IBS, it is necessary to elucidate the pathogenesis of visceral hypersensitivity throughout different life periods.

As the Rome IV criteria highlighted the importance of brain function dysregulation in the generation of visceral hypersensitivity, more attention has been paid to the central mechanism of IBS (Drossman, 2016). The hippocampus, a brain region best known for its role in spatial learning and memory, has recently been found to play an important role in the mental disorders caused by EALs (Gomes et al., 2019). Chronic stress like MS has a great impact on the structure and function of the hippocampus, especially in the brain developing period (McEwen et al., 2016; Katahira et al., 2018). In the investigation of mechanisms of stress, the hippocampus is thought to play the “gateway” role (McEwen et al., 2016). In functional magnetic resonance imaging (fMRI) studies, abnormal activation in the hippocampus was observed in patients with IBS, and the result was verified in IBS-like rat models (Wouters et al., 2012; Mayer et al., 2015). Moreover, Chen et al. verified that the hippocampus was involved in the formation of visceral hypersensitivity in MS adult rats (Chen et al., 2015; Chen et al., 2017). However, the associated mechanism in regulating the formation of visceral hypersensitivity in the hippocampus throughout different life periods remains unclear.

Axon guidance molecules combined with their receptors properly guide the axon to its target to form specific synaptic connections during the development of brain regions, such as the hippocampus (Skutella and Nitsch, 2001; Kim and Kim, 2020). Aberrant expression of the axon guidance system can lead to brain disorders, and thus impact behaviors (Van Battum et al., 2015; Zhang et al., 2021). The Netrin-1, one of the most characterized axon guidance cues, is highly enriched at synapses, especially in CA1 regions of the hippocampus (Glasgow et al., 2018). Netrin-1 could bind to the receptors deleted in colorectal cancer (DCC), neogenin-1(Neo-1), or uncoordinated (UNC5) and then participate in the formation of various chronic pain conditions (Wu et al., 2016; Li et al., 2020; Xiao et al., 2021). It was reported that upregulation of Netrin-1 in the spinal cord enhanced the sprouting and infiltration of afferent nerves and thus led to neuropathic pain (Wu et al., 2016) or osteoarthritis pain (Xiao et al., 2021). In addition, Netrin-1 could promote the maturation of immature or nascent synapses and the formation of long-term potentiation (LTP; Glasgow et al., 2018). It is generally accepted that the enhancement of synapse plasticity is positively related to central sensitization in visceral hypersensitivity (Wang et al., 2015). However, it is unknown about the role of Netrin-1 in the hippocampus in MS-induced visceral hypersensitivity.

In this study, we aimed to explore the molecular mechanism and signaling pathway in the hippocampus underlying MS-induced long-lasting visceral hypersensitivity. Using a combination of RNA-Seq, behavioral tests, lentivirus interference, and molecular biology techniques, we found that over-expression of Netrin-1 could induce visceral hypersensitivity and anxiety disorder in different life periods through activating DCC/GluA1 pathway in the hippocampus.

## Materials and methods

### Animals

**FIGURE 1:**
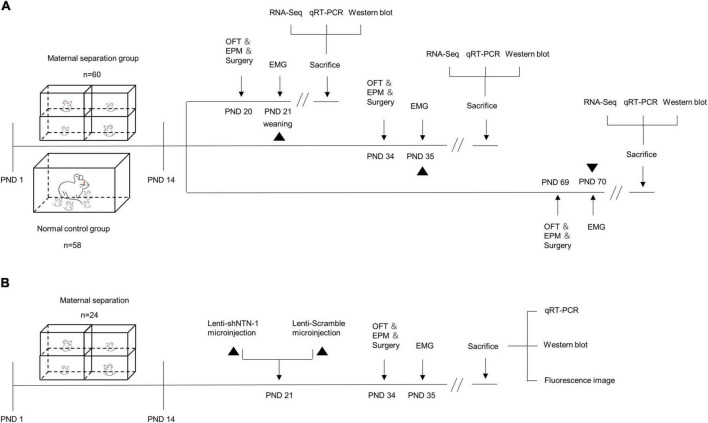
The schematic of experimental procedures. **(A)** Maternal separation (MS) procedures were established on postnatal day (PND) 1–14 (*n* = 60), and the normal control (NC) rats remained undisturbed (*n* = 58). MS and NC rats were randomly subdivided into PND21, PND35, and PND70 groups. The open-field test (OFT), elevated plus maze (EPM) test, and surgery were performed a day before electromyogram (EMG) recording. The visceromotor response (VMR) to colorectal distension (CRD) was recorded by EMG on PND21, PND35, and PND70. After visceral sensitivity was measured, the rats were sacrificed and the hippocampus were dissected for RNA-Seq, quantitative real-time PCR (qRT-PCR), and Western blot. **(B)** MS procedures were established on PND 1–14 (*n* = 24). On PND 21, rats were randomly assigned into two groups and were subjected to anti-Netrin-1 shRNA (Lenti-shNTN-1) or scrambled control shRNA (Lenti-Scramble) microinjection into the CA1 regions of the hippocampus. EMG was recorded on PND35, and the OFT, EPM, and surgery were performed a day in advance. After visceral sensitivity was measured, rats were sacrificed and the hippocampus were dissected for qRT-PCR and Western blot. The accuracy of injection sites was confirmed by fluorescence imaging.

Twenty-three pregnant Wistar rats were purchased from B&K Universal Group Ltd. (Shanghai, China). All pregnant rats were housed separately at a constant temperature with light from 7:00 am to 7:00 pm and had unlimited access to food and water. The day of delivery was recorded as PND0. After giving birth, the mothers were kept together with their newborn offspring to feed and take care of them until PND21. To rule out the effects of the estrous cycle, only male pups were involved in this study (*n* = 157). However, 11 pups died during the MS procedure, and four rats died during the microinjection procedure. Therefore, 142 male pups were finally used for further experiments in this study (study design shown in Figure 1). All animals were treated in accordance with the guidelines for the Care and Use of Laboratory Animals, and all procedures were approved by the Animal Research Committee of the Tongji University, Shanghai, China.

### Animal models

A rat model of MS reported in previous research by the authors was established (Yi et al., 2017). In summary, the pups were randomly assigned to the MS and normal control (NC) groups on PND1. During PND2-PND14, the MS pups were moved gently from their mother and siblings at 10:00 am to separate small plastic boxes (19 cm × 10 cm × 14 cm) on a heating pad (37 ± 0.5°C) in another room, suffering a 3 h-maternal deprivation daily. NC pups were left undisturbed. All pups were weaned and sex-determined on PND21. Both groups of pups were randomly subdivided into post-weaning (PND21), prepubertal (PND35), and adult (PND70) groups separately, which determines the time they would be disposed of. Five to six male rats of the same subgroup were then kept in one cage until the experiments were performed.

### Behavioral tests

Anxiety-like behavioral tests were performed by an observer blinded to the identity of the experimental groups. Each test was performed once on each rat. The results were measured using the EthoVisionXT8.0 Tracking System (Noldus Information Technology, Wageningen, the Netherlands). To adapt to the environment, animals were transferred into the specific testing room 24 h before the actual tests. The floor and the testing apparatus were cleaned with 75% ethanol and dried between two tests.

#### Open-field test

Exploratory activity and anxiety-like behaviors were assessed using an open-field apparatus, which consisted of a black-painted wooden rectangular box (80 cm × 80 cm × 40 cm) and a video camera placed 1 m above it to capture the whole sight of the box. The bottom of the box was divided into a center zone (40 cm × 40 cm) and an outside zone. Each rat was transferred gently to the center of the arena and allowed to move freely for 5 min. Total movement distance, the travel pathway, the frequency of entries into the center zone, and the number of rearing of each rat were recorded.

#### Elevated-plus maze test

The Elevated-Plus Maze (EPM) tests were carried out in an odorless plastic cruciform apparatus 50 cm above the floor, which was formed by a pair of open arms (50 cm × 10 cm), a pair of closed arms (50 cm × 10 cm × 40 cm), and a center platform (10 cm × 10 cm). After adapting in the open field for 5 min, the rat was placed on the center platform with its head facing a specified open arm and started being monitored by a digital camera for 5 min. The travel pathway, the time spent in open or closed arms, the number of entries to open or closed arms, and the anxiety index (anxiety index = 1 - [(time spent in open arms/total time on the maze + number of entries to the open arms/total exploration on the maze)/2]) were recorded for further evaluation of anxiety.

### Visceral sensitivity assessment

Visceral sensitivity was assessed by recording animals’ visceral motor response (VMR) to colorectal distension (CRD), which was described in a previous study (Yi et al., 2017; Yi et al., 2019). When behavioral tests were completed, rats were anesthetized with an intraperitoneal injection of pentobarbital sodium (50 mg/kg), then a pair of Teflon-coated silver wires were implanted in the left obliques externus abdominis 1 day before EMG recordings (study design shown in Figure 1A). The rats were caged separately for rest and fasted overnight before the assessment. On the ensuing visceral sensitivity test day, each rat was placed into a well-sized oval retainer made of Perspex (Yuyan Instruments, Shanghai, China). The EMG signal was recorded by the Amplifier (Brownlee Precision Model 440, United States) and Axon Digidata 1440A data acquisition system (Molecular Devices, United States). A pre-made latex balloon (0.5 cm diameter for rats on PND21, 1 cm diameter for rats on PND35, 2 cm diameter for rats on PND70) tied around a medical catheter (2 mm diameter) was inserted into the colorectal cavity and fixed in place with surgical tape around the tail. The baseline EMG was recorded for the first 20 s. Then gas was injected within 1 s to maintain a certain pressure for the next 20 s. CRD was performed at graded pressures of 40 and 60 mmHg, which was previously proved to be an innocuous stimulus and painful stimulus, respectively (Myers and Greenwood-Van Meerveld, 2012). Each test was repeated three times with a 5-min inter-stimulus interval for recovery. The VMR was quantified by calculating the difference between the area under the curve (AUC) of baseline and the CRD period using Clamp Fit 10.7 (Molecular Devices, United States). AUC was calculated for the 20-s distention period normalized by the 20-s before distention baseline. To compare the change in visceral sensitivity among three different age periods, the relative VMR ratio of each MS group (i.e., the relative AUC/s ratio) was calculated by the mean AUC/s of each MS group normalized by the corresponding NC group (Yi et al., 2017).

### RNA-seq

Thirty minutes after VMR assessment, the rats were quickly sacrificed and a total of 24 rats’ tissues of the hippocampus were dissected under RNase-free conditions. Total RNA was extracted and then quantified and qualified by Agilent 2100 Bioanalyzer (Agilent Technologies, Palo Alto, CA, United States), NanoDrop (Thermo Fisher Scientific Inc.), and 1% agarose gel. One microgram of RNA of each sample was used to construct sequencing libraries. Afterward, RNA-seq was performed using an Illumina HiSeq instrument according to the manufacturer’s instructions (Illumina, San Diego, CA, United States). In the phase of data analysis, each MS group was compared with the age-matched NC group. Due to the small sample size of each group, the absolute fold change ≥ 1.2 accompanied by a *P*-value < 0.05 was considered the threshold to detect differentially expressed genes (DEGs). Pathway enrichment analysis was performed using the Kyoto Encyclopedia of Genes and Genomes (KEGG). The DEGs of the three periods of age were put together to draw a Venn diagram to find the specific genes of rats with visceral hypersensitivity from the post-weaning period to adulthood.

### Quantitative real-time PCR

The expression levels of mRNA were examined by Quantitative real-time PCR (qRT-PCR) and the primers used are listed in Table 1. Briefly, mRNA was extracted and corresponding cDNA was captured through reverse transcription using PrimeScript™ RT Master Mix (TaKaRa). PCR reactions were carried out with TB Green^®^ Premix Ex Taq™ (TaKaRa) and performed on QuantStudio7 Flex (Applied Biosystems). The procedure was repeated three times for each specimen. GAPDH was used as the internal reference, and the 2^–ΔΔ*Ct*^ method was used for data analysis.

**TABLE 1:** List of qRT-PCR primers.

Gene name	Sequence		
	Forward		Reserve
Netrin-1	CCTTCCTCACCGACCTCAACAATC		CTTCTTGCCGAGCGACAGAGTG
DCC	TGCCTCGTCTTGCTGCTGATTG		CTCCTCTTCCTCCTCGTCCTCTTC
Neo-1	TGTGATGGTGACCAAAGGCA		GGAGGCTGCCAGTTCACTATT
Unc5A	CATTTCCGTGCCTGCTGGGTAG		GGTGATCGTGTGTGCCTGAATCC
Unc5B	TTGTGGTTCTGGCAGTTCTCATGG		GTCAGTGATGTCCGTGTCGAAGTC
Unc5C	GCTGAGGTGGAGTGGCTAAAGAATG		CTGAGAGTCGTGCCTGCTTGATG
Unc5D	GCAGGGAAAGTGGGAGGAAGTAATG		AAGCAGCACATGACAGGCGAAG
NR1	TGGTGGCAGATGGCAAGTTTGG		ACGCTCATTGTTGATGGTCAGTGG
NR2A	TCCAGCAGCAAGCCACAGTTATG		TGAAGTCTCGGTAGCCAGGGAAG
NR2B	AGGAGGAAGTAAGACCAGCACAGG		AGGAAGCGGGAGGCAAATGAATG
GluA1	AGTCCAAGCCAGGTGTCTTCTCC		CTCTTCGCTGTGCCATTCGTAGG
GluA2	GCATTTCGGGTAGGGATGGTTCAG		TGGGAGCAGAAAGCATTGGTGAC
PSD95	TCCAGTCTGTGCGAGAGGTAGC		GGACGGATGAAGATGGCGATGG

### Western blot

Protein samples harvested from the hippocampus were lysed by cold lysis buffer (RIPA: PMSF = 100:1) on ice. The concentration of protein was quantified by BCA Protein Assay Kit (Beyotime Biological Co., Ltd.). Twenty micrograms protein samples combined with loading buffer were subjected to SDS-PAGE electrophoresis, transferred onto membranes, and blocked by 5% milk in TBST. Subsequently, the membranes were incubated overnight with primary antibodies, including Netrin-1 (1:500, ab126729, Abcam), DCC (1:1,000, ab273570, Abcam), Neo-1 (1:1,000, 20246-1-AP, Proteintech), GluA1 (1:1,000, ab183797, Abcam), NR1 (1:1,000, ab109182, Abcam), NR2A (1:1,000, ab124913, Abcam), NR2B (1:1,000, ab65783, Abcam), PSD95 (1:1,000, ab36233, Abcam), or Tubulin (1:1,000, ARG62347, Arigo Biolaboratories Co.). On the following day, membranes were incubated with horseradish peroxidase (HRP)-conjugated goat anti-mouse/rabbit IgG antibody (1:5,000, ARG65350/65351, Arigo Biolaboratories Co.) and visualized using an ECL Chemiluminescent Kit (Sigma, United States). Gel image analyses were performed using the ImageJ software.

### Lentivirus interference

The lentiviral vectors used in the current study were pCLenti-U6-shRNA (NTN1)-CMV-EGFP(Lenti-shNTN-1) and pCLenti-U6-shRNA(scramble)-CMV-EGFP (Lenti-scramble), which was purchased from Obio Technology (Shanghai).

Since Netrin-1 is enriched in CA1 dendritic spines of the hippocampus, CA1 was chosen as the injection site (Cembrowski et al., 2016). All experiments investigating the efficiency and effectiveness of Lenti-shNTN-1 were conducted on PND35 owing to the most enhanced visceral sensitivity caused by MS during this specific period. On PND21, rats were anesthetized with an intraperitoneal injection of pentobarbital sodium (50 mg/kg), and then the lentivirus vectors (3 ul, 5 × 10^8^ transducing units [TU]/mL) were slowly microinjected bilaterally at the rate of 0.08 μl/min at the following coordinates: anterior–posterior (AP) –3 mm from the bregma, ± 1.8 mm lateral to midline (L), and 3.5 mm ventrally from the skull surface. After injection, the injector cannula was raised by 0.1 mm and held for 5 min to allow for diffusion before extraction. Both groups of MS rats were kept undisturbed for a 2-week recovery and then delivered to behavioral tests and EMG recording (study design shown in Figure 1B). The tissue of the hippocampus or the whole brain was collected 30 min after EMG recording for PCR, Western blot, and fluorescence image analysis. The injection sites and the spread of the viral infection in MS rats were verified at 40 × magnification using an Olympus BX53 light microscope with the 473-nm laser. For the verification of the virus expression, 40 μm thick sections, spanning the entire dorsal hippocampus from Bregma −1.72 to −4.68 mm, were collected according to the stereotaxic atlas of Paxinos and Watson (Paxinos and Watson, 2006).

### Statistics analysis

The data were reported as mean ± SEM. The normal distribution of parameters and homogeneity of variance was evaluated separately by the Shapiro-Wilk test and Levene test. The comparison between the two groups of data was accomplished using the two-tailed Student’s test. Three-group comparisons were carried out using a one-way ANOVA test followed by the least-significant difference test. All statistical analysis was performed using the SPSS 23.0 software (IBM), and *p*-values lower than 0.05 were considered significant.

## Results

### Visceral hypersensitivity and anxiety-like behaviors caused by maternal separation from the post-weaning period to adulthood

**FIGURE 2:**
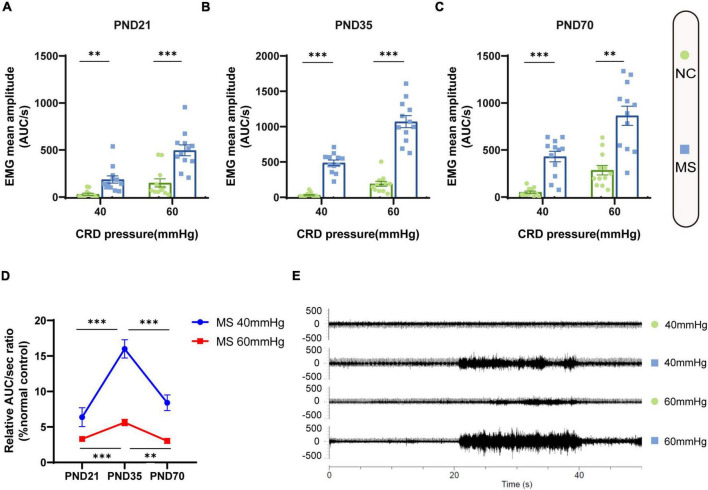
Visceral hypersensitivity occurred in MS rats from the post-weaning period to adulthood and was more pronounced on PND35. **(A–C)** VMR amplitudes to 40 and 60 mmHg CRD on PND21 **(A)**, PND35 **(B)**, and PND70 **(C)** were significantly higher than those of NC rats. On PND21: NC, *n* = 12; MS, *n* = 12. On PND35: NC, *n* = 12; MS, *n* = 12. On PND70: NC, *n* = 12; MS, *n* = 12. All data were given as mean ± SEM. ^**^P < 0.01, ^***^P < 0.001, two-tailed unpaired Student’s *t*-test. **(D)** The relative VMR ratios of MS to NC rats in three different age periods. The relative VMR ratios of MS rats to NC rats on PND35 were significantly higher than that of PND21 and PND70. All data were given as mean ± SEM. ^**^P < 0.01, ^***^P < 0.001, one-way ANOVA test followed by least-significant difference test. AUC/s, the area under the curve per second. **(E)** The representative external abdominal oblique muscle EMG recordings of NC and MS rats.

**FIGURE 3:**
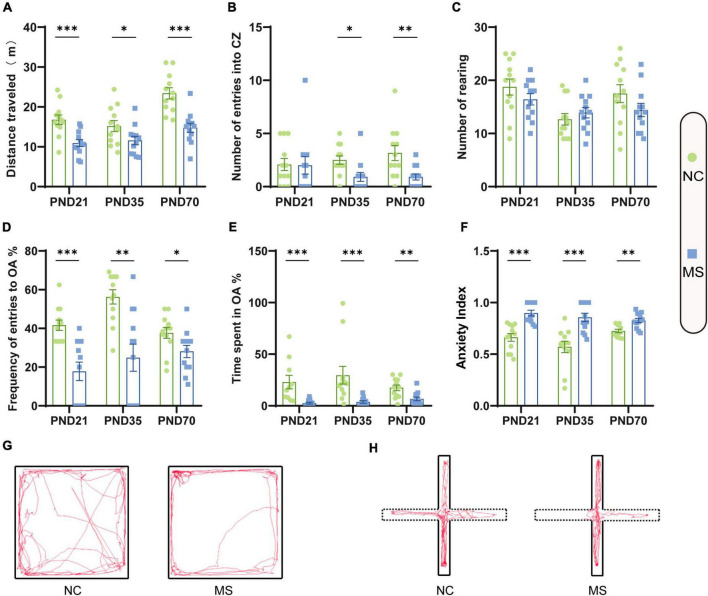
Anxiety-like behaviors were increased in rats of MS groups. Before EMG recordings, all rats were subjected to the OFT test **(A–C)** and EPM test **(D–F)** to evaluate anxiety-like behaviors. **(A)** The distance traveled by MS rats in the open field was significantly less than that of NC rats. **(B)** On PND35 and PND70, MS rats showed less entries into the center zone (CZ). **(C)** There was no significant difference in the number of rearing in the three age periods. **(D)** MS rats on PND21, PND35, and PND70 showed a significantly lower probability of open arms (OA) entries than NC rats. **(E)** MS rats in three groups spent significantly less percentage of time in the OA than NC rats. **(F)** MS rats in three groups showed a significantly higher anxiety index than NC rats. The anxiety index integrated with the EPM behavioral measures was calculated as follows: anxiety index = 1 − [(time spent in OA/total time on the maze) + (number of entries to the OA/total exploration on the maze)/2]. On PND21: NC, *n* = 12; MS, *n* = 12. On PND35: NC, *n* = 12; MS, *n* = 12. On PND70: NC, *n* = 12; MS, *n* = 12. All data were given as mean ± SEM. *P < 0.05, ^**^P < 0.01, ^***^P < 0.001, two-tailed unpaired Student’s *t*-test. **(G)** Representative animal track in the OFT and **(H)** EPM.

Visceral sensitivity was assessed on PND21, PND35, and PND70 (Figure 2), and the typical EMG images were presented (Figure 2E). VMRs of MS rats to both 40 and 60 mmHg CRD stimulation were significantly higher than NC ones in three different age periods, which indicated that MS could induce visceral hypersensitivity in rats from the post-weaning period to adulthood (Figures 2A–C). The relative VMR ratio in MS rats among three age periods was compared. It was found the relative VMR ratio was significantly higher on PND35 than that on PND21 or PND70, indicating that the visceral hypersensitivity induced by MS reached a peak before adulthood (Figure 2D). The anxiety-like behaviors were observed by Open-Field Test (OFT) and EPM (Figure 3). The total traveled distance of MS rats during OFT was shorter than NC rats in all three age periods. Furthermore, MS rats had less entries into the center zone on PND35 and PND70, while no significant difference was shown in the number of rearing (Figures 3A–C). As for the EPM test, MS rats showed less percentage of frequency of entry into open arms and less percentage of time spent in them in all three age periods compared with the NC ones (Figures 3D,E). Moreover, the anxiety index that integrates the EPM behavioral measures also exhibited increased anxiety-like behaviors in MS rats from the post-weaning period to adulthood (Figure 3F). Representative animal track in OFT (Figure 3G and Supplementary Figures 1A–C) and EPM (Figure 3H and Supplementary Figures 1D–F) showed the obvious difference between MS and NC rats.

### Identification of differentially expressed genes in the hippocampus of maternal separation rats through RNA-seq

**FIGURE 4:**
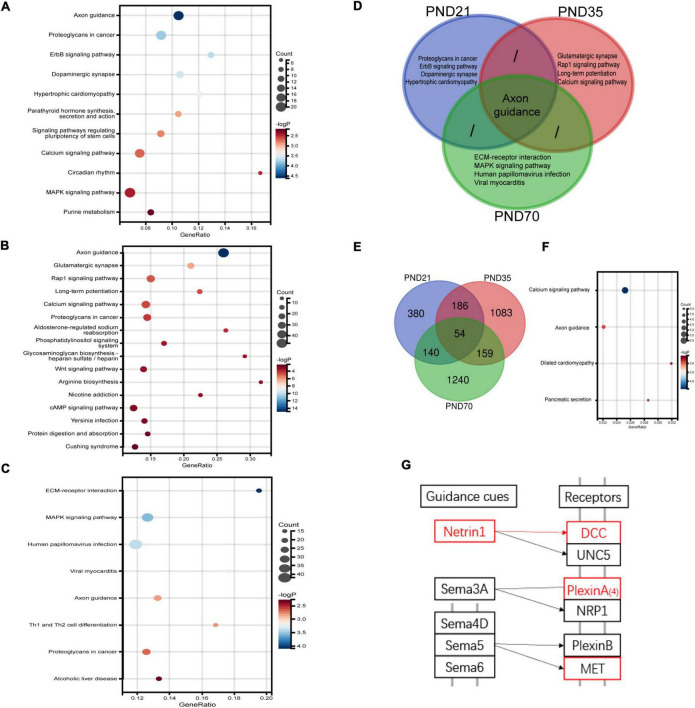
RNA-seq results of the hippocampus in MS and NC rats of three different age periods (*n* = 4 per group). **(A–C)** The differentially expressed genes (DEGs) on PND21 **(A)**, PND35 **(B)**, and PND70 **(C)** were significantly enriched in various KEGG signaling pathways. The top-ranked pathways were visualized with bubble plots. **(D)** Venn diagram of enriched pathways showed that axon guidance pathway was the only shared enriched pathway which ranked among top five of all age periods. **(E)** Venn diagram of DEGs in the hippocampus showed that 54 DEGs were shared in different age periods. **(F)** A total of 54 shared DEGs were significantly enriched in various pathways, including the axon guidance pathway. **(G)** A simplified schematic of the axon guidance pathway. Netrin-1, its specific receptor deleted in colorectal cancer (DCC), as well as other two molecules marked in red were upregulated among MS rats of three periods. Netrin-1 and DCC were specific ligands or receptors for each other.

RNA-Seq was used to explore the specific pathways and genes in the hippocampus involved in the pathological process of visceral hypersensitivity induced by MS in three age periods (Figure 4). KEGG analysis revealed significant enrichment of genes in various pathways (Figures 4A–C and Supplementary Figure 2). Particularly, the axon guidance pathway ranked first on both PND21 and PND35, and also ranked high on PND70. Furthermore, the axon guidance pathway seemed to be the only shared pathway in the top five enriched KEGG pathways of all age periods (Figure 4D). Volcano plots illustrated the distribution and variation of upregulated and downregulated DEGs in different periods (Supplementary Figure 3). To further explore the shared DEGs in different periods, a Venn diagram was drawn, which detected a total of 54 DEGs (Figure 4E). Among the shared DEGs, four axon guidance-related pathway genes were identified, including Netrin-1, DCC, PlexinA4, and MET, which were upregulated in MS rats of different periods (Figures 4F–G). Interestingly, Netrin-1 and DCC were specific ligands or receptors for each other, indicating that their co-upregulation might be a potential mechanism in MS-induced visceral hypersensitivity.

### Abnormalities of Netrin-1 and related signaling pathway in the hippocampus of maternal separation rats

**FIGURE 5:**
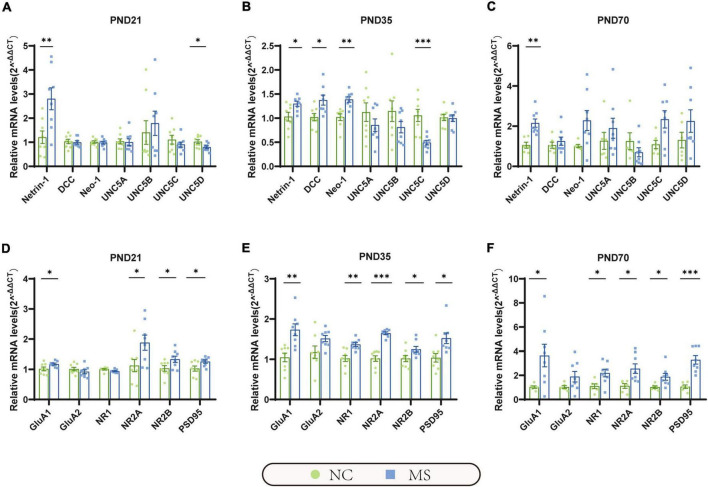
The mRNA expression of Netrin-1 and GluA1 were significantly increased in the hippocampus in MS rats of three different age periods. **(A)** On PND21, the mRNA expression of Netrin-1 was significantly increased, but its receptor uncoordinated (UNC5D) was significantly decreased. **(B)** On PND35, the mRNA expression of Netrin-1, and its receptor DCC, neogenin-1 (Neo-1) were significantly increased, but UNC5C was significantly decreased. **(C)** On PND70, the mRNA expression of Netrin-1 was significantly increased, but its relative receptors showed no significant upregulation. **(D–F)** GluA1, NR2A, NR2B, and postsynaptic density 95 (PSD95) were significantly increased in all three MS groups. Besides, NR1 was found upregulated in MS rats on PND35 **(E)** and PND70 **(F)**. On PND21: NC, *n* = 8; MS, *n* = 8. On PND35: NC, *n* = 8; MS, *n* = 8. On PND70: NC, *n* = 6; MS, *n* = 8. All data were given as mean ± SEM. *P < 0.05, ^**^P < 0.01, ^***^P < 0.001, two-tailed unpaired Student’s *t*-test.

**FIGURE 6:**
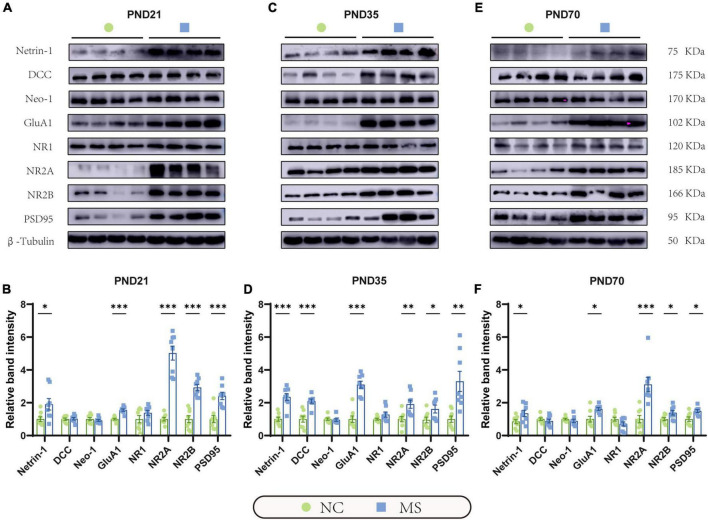
Netrin-1 and GluA1 protein were upregulated in the hippocampus in rats of three MS groups. Representative immunoblots showed the expression of Netrin-1, DCC, Neo-1, GluA1, NR1, NR2A, NR2B, PSD95, and β-Tubulin on PND21 **(A)**, PND35 **(C)**, and PND70 **(E)**. **(B)** Western blot analysis showed increased expression of Netrin-1, GluA1, NR2A, NR2B, and PSD95 in the hippocampus in MS rats on PND21, **(D)** PND35, and **(F)** PND70. Furthermore, the protein expression level of DCC was upregulated on PND35. On PND21: NC, *n* = 8; MS, *n* = 8. On PND35: NC, *n* = 8; MS, *n* = 8. On PND70: NC, *n* = 8; MS, *n* = 8. All data were given as mean ± SEM. *P < 0.05, ^**^P < 0.01, ^***^P < 0.001, two-tailed unpaired Student’s *t*-test.

qRT-PCR (Figure 5) and Western blot analysis (Figure 6) were performed to verify the results from RNA-seq at the mRNA and protein levels. Netrin-1 and its receptors DCC, as well as the DCC paralogue Neo-1 and UNC5 families (UNC5A-D), were analyzed. The results showed that both mRNA and protein of Netrin-1 were upregulated from the post-weaning period to adulthood in MS rats (Figures 5A–C, 6). However, the mRNA of DCC and Neo-1 were found upregulated only on PND35, and no significant upregulation of mRNA was seen in UNC5 families. Thus, DCC and NEO-1 were further measured at the protein level in three age periods. The results showed that DCC instead of NEO-1 increased in MS rats on PND35, while none of them altered on PND21 or PND70 when compared with the NC ones. The specific upregulation of DCC provided a profound explanation for the most severe visceral hypersensitivity on PND35.

LTP in the hippocampus has been generally recognized as a trigger in mediating MS-induced visceral hyperalgesia (Chen et al., 2015; Chen et al., 2017), and Netrin-1 has been proved to be a critical factor in enhancing hippocampal LTP (Glasgow et al., 2018). AMPA receptors (AMPARs) and NMDA receptors (NMDARs) are major contributors to the formation of hippocampal LTP (Kullmann and Asztely, 1998; Peters et al., 2021). Therefore, the mRNA and protein expressions of hippocampal AMPARs (mainly comprised of GluA1 and GluA2 subunits; Diering and Huganir, 2018) and NMDARs (including predominant functioning subunits NR1, NR2A, and NR2B; Hansen et al., 2017) in the hippocampus were further evaluated. qRT-PCR results showed that the gene expression of GluA1, NR2A, and NR2B of MS rats were significantly higher than the NC ones from PND21 to PND70, whereas NR1 was upregulated only on PND35 and PND70, and no difference was detected in GluA2 (Figures 5D–F). The results of the Western blot also revealed the increase of GluA1 as well as NR2A and NR2B in MS rats from the post-weaning period to adulthood (Figure 6). In addition, the expression of PSD95, as the marker of the postsynaptic density, was found to increase in MS rats in both mRNA and protein levels compared to NC rats (Figures 5D–F, 6).

### Knockdown of hippocampal Netrin-1 in the post-weaning period reversed visceral hypersensitivity and anxiety-like behaviors in the later phase of life

**FIGURE 7:**
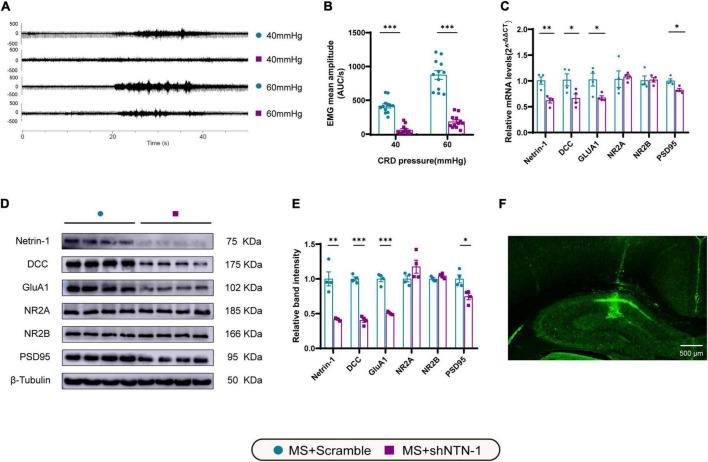
Knockdown of Netrin-1 (NTN-1) in hippocampal CA1 regions decreased not only visceral sensitivity but also the expression levels of mRNA and proteins of Netrin-1, DCC, GluA1, and PSD95. **(A)** The representative external abdominal oblique muscle EMG recordings. **(B)** VMR amplitudes to 40 and 60 mmHg CRD were significantly reduced in rats expressing Lenti-shNTN-1 (MS + shNTN-1) than in rats injected Lenti-Scramble (MS + Scramble). MS + Scramble: *n* = 12. MS + shNTN-1: *n* = 12. All data were given as mean ± SEM. ^***^P < 0.001, two-tailed unpaired Student’s *t*-test. **(C)** The mRNA and **(E)** protein expressions of Netrin-1, DCC, GluA1, and PSD95 in the hippocampus were significantly reduced in the MS + shNTN-1 group. **(D)** Representative immunoblots showed the expression of Netrin-1, DCC, GluA1, NR2A, NR2B, PSD95, and β-Tubulin. **(F)** A representative fluorescence image confirmed the successful GFP expression in shNTN-1 infected cells in the hippocampus. Scale bar, 500 μm. MS + Scramble: *n* = 4. MS + shNTN1: *n* = 4. All data were given as mean ± SEM. *P < 0.05, ^**^P < 0.01, ^***^P < 0.001, two-tailed unpaired Student’s *t*-test.

To see if Netrin-1 in the hippocampus is essential for the development of MS-induced visceral hyperalgesia, Lenti-shNTN-1 or Lenti-Scramble were microinjected into the bilateral CA1 regions of the hippocampus of MS rats. Due to the limited tolerance of little pups to the surgery, we chose lentivirus intervention on PND21 rather than earlier. And as mentioned above, visceral hypersensitivity reached its peak on PND35, therefore PND35 was the time point to observe related changes after intervention. The results revealed that the expression levels of Netrin-1 mRNA and protein were considerably inhibited on PND35 after hippocampal Lenti-shNTN-1 administration (Figures 7C–E).

**FIGURE 8:**
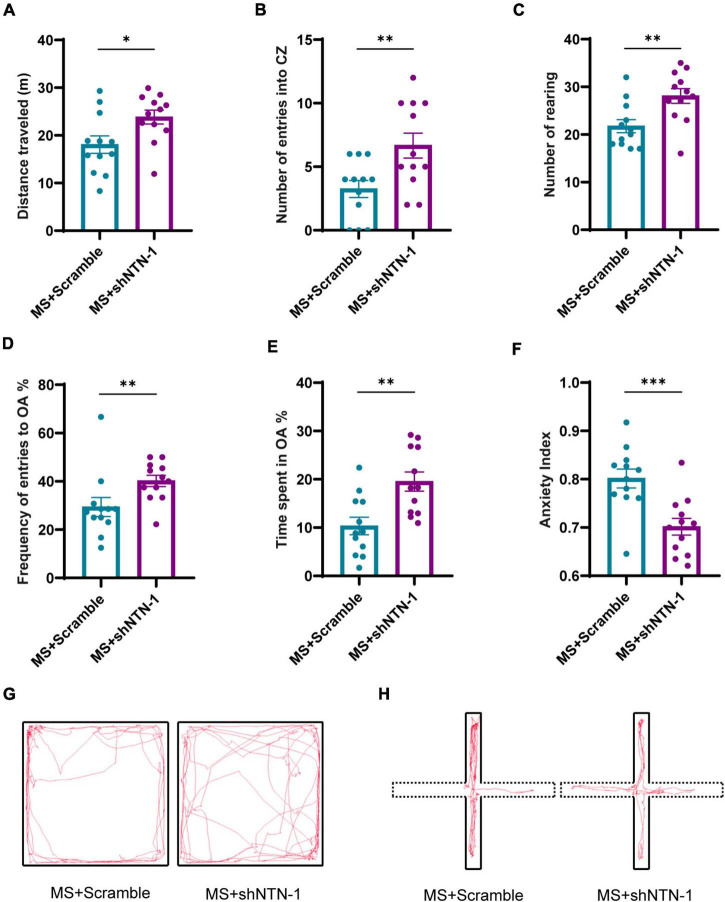
Knockdown of Netrin-1 (NTN-1) in hippocampal CA1 regions reversed the increase of anxiety-like behaviors caused by MS. **(A)** In OFT, compared with rats injected Lenti-Scramble (MS + Scramble), rats expressing Lenti-shNTN-1 (MS + shNTN-1) showed longer distance traveled, **(B)** more entries into the center zone, and **(C)** more rearing. **(D)** In the EPM test, the percentage of numbers of entries into OA and **(E)** the percentage of time spent in OA in the MS + shNTN-1 group were significantly increased, and **(F)** the anxiety index was reduced significantly. **(G)** Representative animal track in the OFT and **(H)** EPM. MS + Scramble: *n* = 12. MS + shNTN1: *n* = 12. All data were given as mean ± SEM. *P < 0.05, ^**^P < 0.01, ^***^P < 0.001, two-tailed unpaired Student’s *t*-test.

The most remarkable finding was that the VMRs to both 40 and 60 mmHg CRD stimulations were deeply compromised on PND35 by hippocampal knockdown of Netrin-1 (Figures 7A,B). The results of behavioral tests also showed the therapeutic effect of Netrin-1 knockdown in aversive emotion. Compared with rats expressing Lenti-Scramble, rats expressing Lenti-shNTN-1 showed more rearing, more entries to center zones, and longer traveled distance in OFT (Figures 8A–C,G). Besides, the percentage of the number of entries into open arms and the percentage of time spent in them were increased while the anxiety index was reduced significantly in EPM after the knockdown of Netrin-1 (Figures 8D–F,H).

### Knockdown of Netrin-1 inhibited the DCC/GluA1 signaling pathway in the hippocampus

To verify the downstream signaling pathway of Netrin-1 in the process of visceral hyperalgesia, the genes and proteins that were previously found to be upregulated accompanied by Netrin-1 in MS rats were examined again (Figures 7C–E). Notably, a significant reduction in the mRNA and protein expression of DCC and GluA1 after administration of Lenti-shNTN-1 was observed. The hippocampal level of PSD95 exhibited a slight decrease after Netrin-1 knockdown. However, Lenti-shNTN-1 failed to influence the expression of NR2A and NR2B. The representative fluorescence image confirmed the successful GFP expression in lentiviruses infected cells in the hippocampus (Figure 7F and Supplementary Figure 4).

## Discussion

In this work, the molecular mechanism underlying the continuous visceral hypersensitivity in different life periods caused by MS was investigated for the first time. Netrin-1 was identified as a key molecular cue whose long-lasting upregulation contributed to the development of visceral hypersensitivity and anxiety-like behaviors from the post-weaning period to adulthood. Moreover, the results suggested that Netrin-1/DCC/GluA1 signaling pathway may be a potential therapeutic target for the treatment of visceral hypersensitivity and associated anxiety disorder.

EALs have been recognized as common causes of refractory IBS in adult patients (Liu et al., 2017). MS, as a strong early life stressor, has been used as a well-established rodent model to explore stress-induced visceral hyperalgesia (Riba et al., 2018). In this study, visceral hypersensitivity induced by MS was observed to occur during the post-weaning period. Notably, a steep trend line about the change of visceral sensitivity was also observed when comparing VMRs among MS groups of different age periods. The results showed that visceral sensitivity was the most enhanced in the prepubertal period. A recent clinical survey focusing on the correlation between EALs and gastrointestinal symptoms also revealed that the largest effects of EALs on gastrointestinal distress happened in late childhood (Callaghan et al., 2020), providing side evidence for the results in this study. Therefore, it seems that visceral hypersensitivity in the prepubertal period, one of the most important mental development periods, should be paid more attention to.

Mental health problems are highly comorbid with visceral hypersensitivity in those who ever experienced EALs (Hendrix et al., 2022). It was reported that the rate of anxiety was five times higher in IBS patients compared to normal individuals and anxiety sufferers were more vulnerable to IBS in return (Lee et al., 2009; Mak et al., 2012). Therefore, researchers have been widely concerned about the relationship between anxiety disorder and IBS. Similar to previous clinical findings, anxiety-like behaviors increased in MS rats. Most importantly, this study proved that visceral hypersensitivity accompanied by anxiety disorder also existed since the post-weaning period for the first time. These findings suggested that early identification of typical gastrointestinal and related psychological symptoms that emerged in the preadult period is essential for the prevention and management of EALs-induced long-lasting IBS.

Moreover, the specific molecular mechanism underlying the phenotypes in the hippocampus was investigated to identify the therapeutic targets. The most significant finding of this study is the identification of Netrin-1, one of the most characterized members of the axon guidance cues, as a key contributor to the early-emerged and long-lasting visceral hypersensitivity induced by MS. Netrin-1 organizes neural network connectivity *via* regulating axon arborization, dendritic growth, and synapse formation, whose function might be sensitive to negative environmental events (Manitt et al., 2009; Chang and Grace, 2018; Torres-Berrio et al., 2020). In addition, Netrin-1 was previously proved to be a major contributor to both neuropathic pain and psychiatric conditions like depression (Li et al., 2020; Torres-Berrio et al., 2020). However, the role of Netrin-1 in visceral hypersensitivity has never been reported. In this study, Netrin-1 was found significantly upregulated in the hippocampus of MS rats during different age periods. After knocking-down hippocampal Netrin-1 in the post-weaning period, the visceral sensitivity and anxiety-like behaviors of MS rats recovered to a great extent on PND35, a period supposed to reach the peak of visceral hypersensitivity according to the results in this work. Overall, continuous upregulation of Netrin-1 was a critical determinant in the development of MS-induced visceral hypersensitivity. Early intervention targeting Netrin-1 in the hippocampus was effective in the treatment of comorbid visceral hypersensitivity and anxiety disorder.

Netrin-1 exhibits different functions depending on the activation of its two main receptor families, DCC and UNC5. In this study, examinations targeting the receptors of Netrin-1 additionally detected the upregulation of DCC in MS rats only on PND35, which was consistent with the most enhanced visceral hyperalgesia in the same period. In addition, the knockdown of Netrin-1 further led to the recovery of the DCC level on PND35. Early studies have proved that the increased formation of synaptic connection caused by Netrin-1 overexpression could be suppressed by the absence of DCC (Kolodziej et al., 1996). In 2020, Torres-Berrio et al. have proposed that the Netrin-1/DCC pathway primes the central nervous system to disruption by stress, which renders individuals more susceptible to develop abnormalities, such as chronic pain and psychiatric conditions (Torres-Berrio et al., 2020). In 2021, Khoury et al. (2021) identified DCC as a gene closely related to chronic pain conditions by genome-wide analysis. These findings indicated that DCC was the key molecule in the process of Netrin-1 regulating visceral hypersensitivity.

It was reported that Netrin-1 and DCC are enriched in dendritic spines of pyramidal neurons in CA1 regions of the hippocampus (Cembrowski et al., 2016; da Silva et al., 2018; Glasgow et al., 2021). Glasgow et al. (Glasgow et al., 2018) demonstrated that increased Netrin-1 binding to DCC was sufficient to trigger LTP in the hippocampus via the insertion of GluA1-containing AMPARs. Some studies have emphasized the critical role of LTP in the hippocampus in the development of somatic hyperalgesia in chronic pain conditions (Chen et al., 2015; Chen et al., 2017). To test whether the Netrin-1/DCC pathway in the hippocampus of MS rats played its role in visceral hypersensitivity *via* such mechanisms as shown above, the expression levels of AMPARs were examined. GluA1 was verified to be upregulated as the increase of Netrin-1. Furthermore, after knocking-down the endogenous protein expression of Netrin-1, the GluA1 was significantly downregulated as well. PSD95, which was strongly correlated with the strengthening of LTP (Zhang and Lisman, 2012) and could be recruited by Netrin-1 (Glasgow et al., 2021), was overexpressed in the hippocampus in all groups of MS rats. As expected, the hippocampal level of PSD95 was decreased after Netrin-1 knockdown, providing evidence for the weakening of LTP. Altogether, a novel hypothesis emerged that the activation of the Netrin-1/DCC/GluA1 pathway, which resulted in strengthening hippocampal LTP in MS rats, finally led to the visceral hypersensitivity and anxiety-like behaviors from the post-weaning period to adulthood.

Both AMPARs and NMDARs are important for the formation of LTP, and Netrin-1 is especially required for NMDA-dependent LTP (Kullmann and Asztely, 1998), the expressions of NMDARs were also examined in this study. Previous studies have stated that NR2A and NR2B are activating agents to promote Netrin-1 secretion from excitatory neurons (Glasgow et al., 2018), and both of them were found upregulated continuously in MS rats in this work. Thus, it was hypothesized that NR2A and NR2B were upstream molecules of Netrin-1 in MS-induced visceral hypersensitivity. However, knocking-down Netrin-1 failed to alter the expressions of NR2A and NR2B of MS rats significantly as a negative feedback regulation. Therefore, the role of NMDARs in MS-induced visceral hypersensitivity needed to be further explored.

Besides, as we know, many other ways such as water avoidance stress and intraperitoneal injection of chicken egg albumin, etc., can also induce visceral hypersensitivity. However, whether the hippocampal Netrin-1 pathway is involved in regulating the visceral hypersensitivity caused by the above non-MS methods needs to be further clarified.

A limitation of this study is the lack of exploration of the role of Netrin-1 in the original formation of MS-induced visceral hyperalgesia. Thus, a hippocampal Netrin-1 specific-knockout rat model should be constructed, and microinjection experiments of recombinant protein of netrin-1 should be accomplished for the next step.

In summary, aberrant upregulation of Netrin-1 contributes to visceral hypersensitivity and anxiety-like behaviors after MS *via* modulating the recruitment of GluA1 in the hippocampus from the post-weaning period to adulthood. In addition, visceral hypersensitivity is observed to vary with time and have a climax in the prepubertal period, which was probably led by the accompanied upregulation of DCC. This work highlights the severer visceral hypersensitivity represented in preadult life, calling for more attention to pediatric IBS. Moreover, it renders the possibility of a novel therapeutic target on the signaling of Netrin-1/DCC/GluA1, which could provide substantial benefit to patients with IBS induced by EALs in clinics.

## Data availability statement

The datasets presented in this study can be found in online repositories. The names of the repository/repositories and accession number(s) can be found in the article/Supplementary material. The RNA-Seq data used in this study have been deposited in the NCBI’s Sequence Read Archive (SRA) (SRA study accession code, PRJNA838219).

## Ethics statement

The animal study was reviewed and approved by Committee on Use and Care of Animals at Tongji University (the ethics approval number: TJAA07820201).

## Author contributions

JW and GD contributed to the conception of the study, performed the experiments, collected the data, performed the data analyses, and wrote this manuscript. TZ and ZD helped perform the experiments, collected the data, and performed the data analyses. YZ and YC collected the data and helped perform the analysis with constructive discussions. SX and HS contributed to the conception of the study and revised the manuscript. All authors contributed to the article and approved the submitted version.

## Conflict of interest

The authors declare that the research was conducted in the absence of any commercial or financial relationships that could be construed as a potential conflict of interest.

## Publisher’s note

All claims expressed in this article are solely those of the authors and do not necessarily represent those of their affiliated organizations, or those of the publisher, the editors and the reviewers. Any product that may be evaluated in this article, or claim that may be made by its manufacturer, is not guaranteed or endorsed by the publisher.
