# Supplementary material for: Upregulation of Netrin-1 in the hippocampus mediates the formation of visceral hypersensitivity induced by maternal separation
Source: Front Mol Neurosci. 2022 Jul 28;15:908911. doi: 10.3389/fnmol.2022.908911 (PMC9366914; doi:10.3389/fnmol.2022.908911)
Supplement: Supplementary file 1 [file Presentation_1.PDF]

Supplementary Material

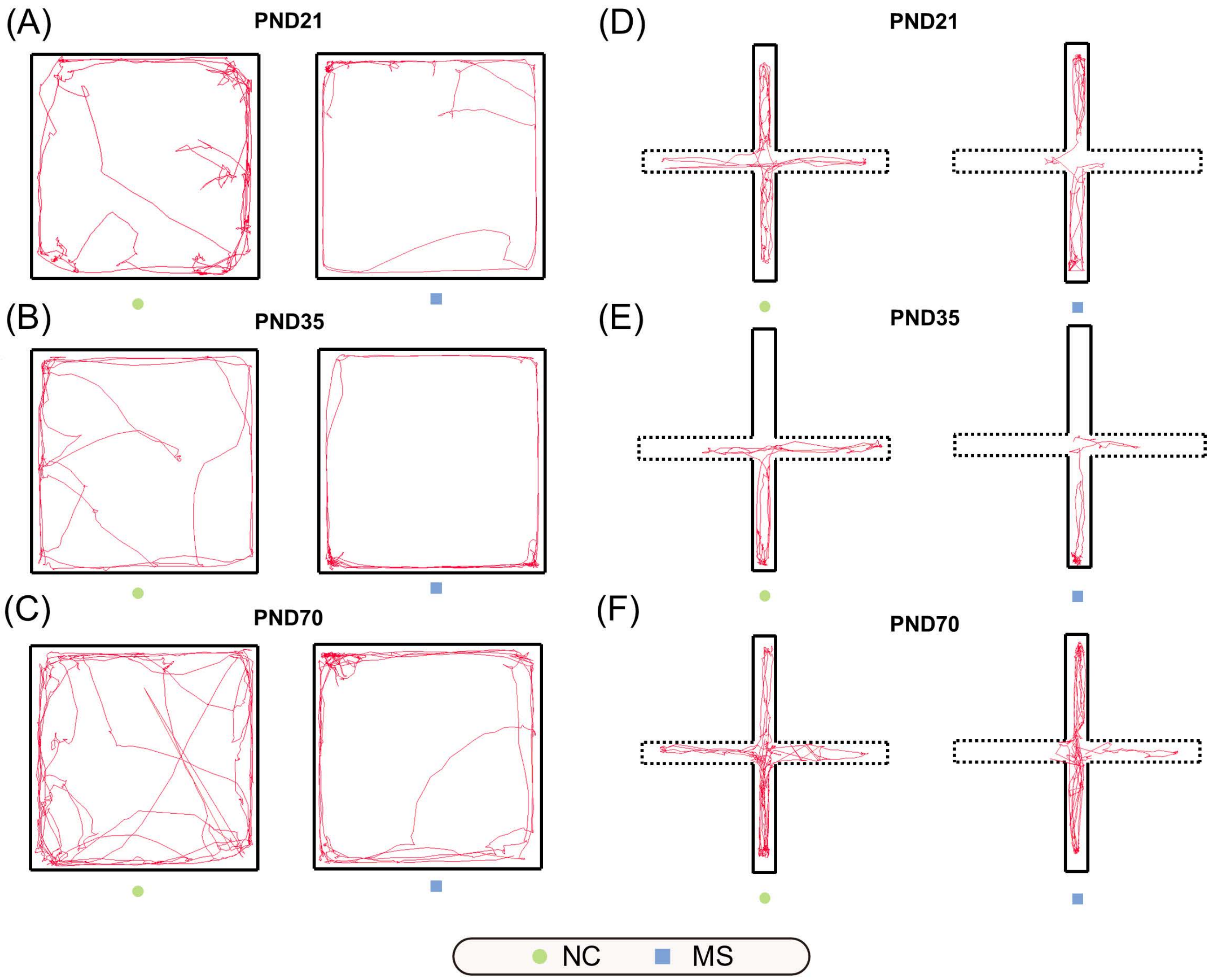

Figure S1. Representative animal track in the open field chamber (A-C) and elevated plus maze (D-F) on PND21(A, D), PND35(B, E), PND70(C, F).

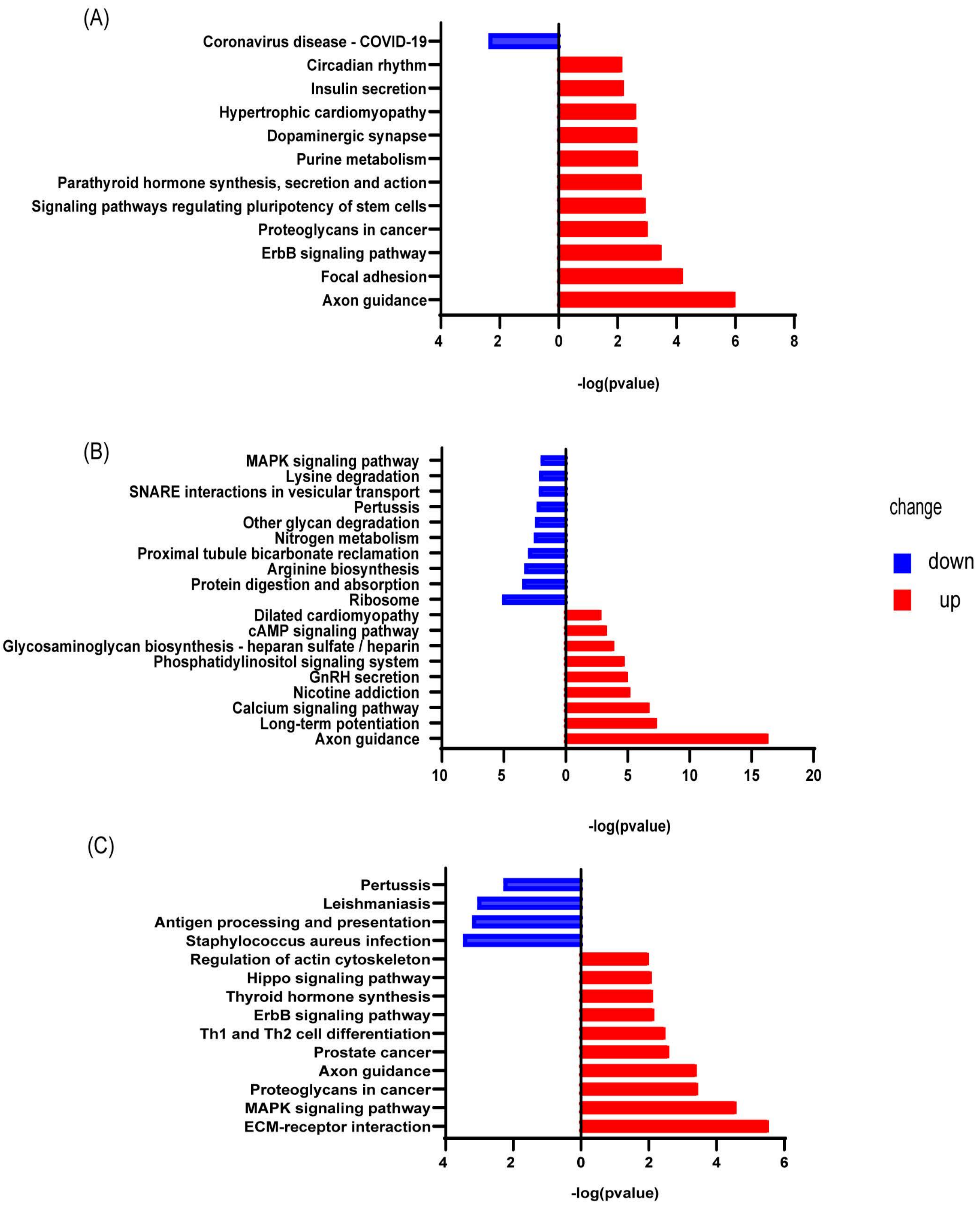

Figure S2. KEGG enichment analysis of the upregulated and downregulated genes separately on 21D (A) ,35D (B) and 70D (C). Axon guidance pathway ranked top 5 in upregulated pathways in all 3 age periods.

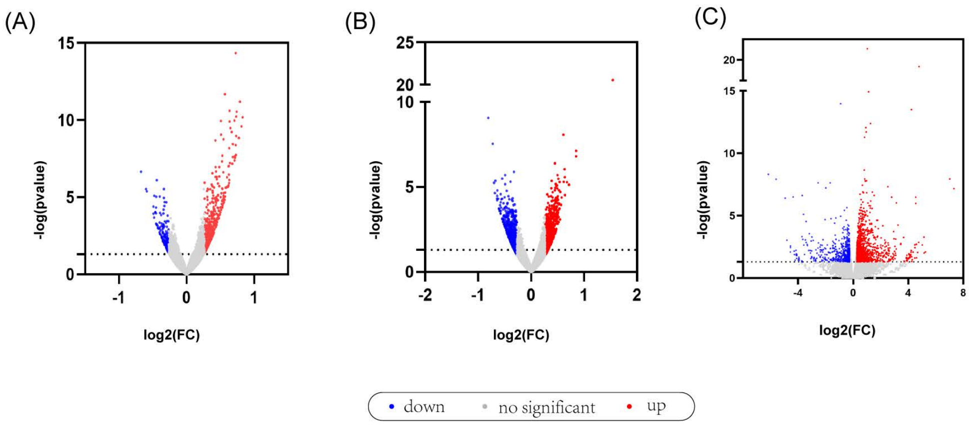

Figure S3. Volcano plots illustrated the distribution and variation of upregulated and downregulated DEGs on PND21(A), PND35(B), PND70(C).

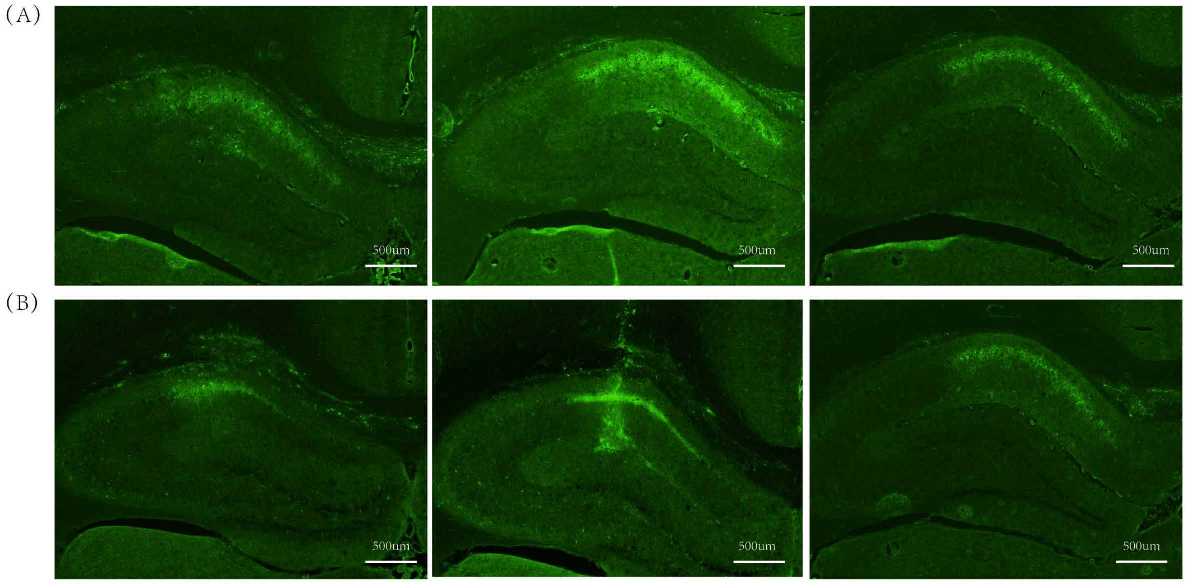

Figure S4. Schematic representation of the average extent of the hippocampus in MS + Scramble group (A) and MS+shNTN-1 group (B).
